# Comparison of two clinical approaches based on visual criteria for secondary caries assessments and treatment decisions in permanent posterior teeth

**DOI:** 10.1186/s12903-022-02112-6

**Published:** 2022-03-18

**Authors:** Cacia Signori, CaCIA collaborative group, Ana Beatriz L. Queiroz, Alessandra B. Avila, Bruna O. Souza, Cácia Signori, Camila R. Dias, Camila T. Becker, Eduardo T. Chaves, Eugênia C. Malhão, Elenara F. Oliveira, Juliana L. S. Uehara, Fernanda G. Silva, Fernanda S. Silva, Gabriel V. L.Kucharski, Gabriele R. Santos, Julia M. Torres, Karoline V. A. Pinto, Laura L. Morel, Leonardo B. Weymar, Marcelo P. Brod, Maria Fernanda Gamborgi, Maximiliano S. Cenci, Renata U. Posser, Thaís S. Vieira, Vitor Henrique Digmayer Romero, Wagner S. Nolasco, Wagner M. S. Leal, Juliana Lays Stolfo Uehara, Vitor Henrique Digmayer Romero, Bruna Lorena Pereira Moro, Mariana Minatel Braga, Fausto Medeiros Mendes, Maximiliano Sérgio Cenci

**Affiliations:** 1 Graduate Program in Dentistry, School of Dentistry, Federal University of Pelotas, Rua Gonçalves Chaves, 457, Pelotas, RS 96015-560 Brazil; 2 Department of Restorative Dentistry, School of Dentistry, Uniavan University Center, Av. Marginal Leste, 3600, Balneário Camboriú, SC 88339-125 Brazil; 3 School of Dentistry, Federal University of Pelotas, Rua Gonçalves Chaves, 457, Pelotas, RS 96015-560 Brazil; 4 Department of Pediatric Dentistry, School of Dentistry, University of São Paulo, Avenida Prof. Lineu Prestes, 2227, São Paulo, SP 05508-000 Brazil

**Keywords:** Caries detection, Dental caries, Restorations, Visual inspection, Secondary caries

## Abstract

**Background:**

This cross-sectional study aimed to compare two clinical approaches based on visual criteria for secondary caries assessments and treatment decisions in permanent posterior teeth.

**Methods:**

The two clinical visual criteria tested for the assessments of restored teeth were: FDI criteria—based on the caries presence, marginal adaptation and staining criteria, adapted from the FDI (International Dental Federation) criteria and CARS criteria—"Caries Associated with Restorations or Sealants" (CARS) criteria described by the International Caries Classification and Management System. Adults were randomized according to the criteria. One calibrated examiner assessed the restorations and assigned the treatment according to the criteria. The primary outcome was replacement indication.

**Results:**

A total of 185 patients were included, totalling 718 restorations. The strongest correlation founded between the methods was for the presence of caries lesions (Rho = 0.829). A moderate correlation (Rho = 0.420) was founded between the treatment decisions proposed by the CARS and by the FDI criteria. The multilevel regression analysis showed that the FDI criteria indicated five times more replacements when compared to the CARS (< 0.001). Also, using the FDI criteria restorations were 2.7 times more related to caries around restorations (p < 0.001) compared to the other criterion.

**Conclusions:**

The visual criteria used on the restoration's assessment directly influences the treatment decision to intervene or not on the restoration. The use of a minimally invasive based approach for assessing secondary caries may prevent overtreatment.

**Supplementary Information:**

The online version contains supplementary material available at 10.1186/s12903-022-02112-6.

## Background

The restoration replacement results on the loss of sound tooth structure, and may cost, at some point, the tooth loss. This process is known as the "death spiral" [1] or repetitive restorative cycle [2, 3]. The replacement of restorations is a standard procedure performed at the dental office [4], corresponding to more than half of the interventions in restorative dentistry [5]. The leading cause of restorations replacements is the presence of caries lesions around the restorations, also known as secondary caries [6–8]. Secondary or recurrent caries has been defined as "lesions at the margins of existing restorations" or "caries associated with restorations or sealants" (CARS) [9]. Secondary caries is a complex process, combining well-known causes of "conventional" caries with the specificities of the restorations and restorative materials [10]. From these specificities, the buffering capability of dental materials, as well as the presence of marginal defects in the restorations, are known to play a role in the process [10]. But in the end, the process results in (and should the diagnosed as) demineralization detectable at the margin of an existing restoration, and the treatment decision should be based solely on the clinical signs observed.

The management of caries lesions around the restorations was considered as one of the critical points needing improvement in dentistry over the next 20 years [11]. The assessment of the restored tooth is a crucial step to allow a proper judgment and treatment decision of old restorations [12]. Whereas other tools may be applied [10], the visual inspection is the most used method to detect secondary caries. However, there is still heterogeneity in the clinical approach used in the assessment by dentists on the visual detection [13]. This may be attributed to some aspects, such as the presence of a gap in the tooth-restoration interface, tooth tissue discoloration and marginal staining around the restoration, and even to the location of the lesion, sometimes in areas difficult to assess, as the approximal surfaces [14]. Moreover, dentists usually mistake some aspects related to the marginal degradation of restorations, such as marginal staining or marginal contour defects, with caries lesions [15–17], even in a scenario where the current knowledge states clearly that what should be identified and reacted to are the signs of demineralization, and more specifically, cavitation [12]. The most recent scientific evidence supports the minimally invasive approach to managing caries around restorations [4, 18].

Therefore, it is necessary to investigate the effect of the detection of secondary caries using different approaches on the dental care treatment decision framework, considering that overdiagnosis can induce overtreatment on restored permanent teeth, as recently shown for primary teeth [19]. Thus, this study aimed to compare two clinical approaches based on visual criteria for secondary caries assessments and treatment decisions in permanent posterior teeth. The hypothesis tested was that the choice of an index system focused on the detection of caries lesions around restorations, instead of criteria focused on the detection of small marginal defects, not clinically relevant, induces fewer restorative interventions in permanent teeth.

## Methods

The STrengthening the Reporting of OBservational studies in Epidemiology (STROBE) guideline [20] was used to report this manuscript (Additional file 1).

### Study design

This cross-sectional study compared two index systems based on visual clinical criteria for the assessments of restored teeth: (1) FDI criteria—based on 3 parameters from the FDI (International Dental Federation) system—caries presence, marginal adaptation and marginal staining; and (2) CARS criteria—"Caries Associated with Restorations or Sealants" (CARS) criteria described by the International Caries Classification and Management System (ICCMS). Adults were randomized according to the assessment criteria. One trained examiner assessed the surfaces using both criteria to minimize other sources of variations differenf from the systems by themselves. After the diagnosis according to the sorted criterion and establishment of the treatment decision by the examiner, the same restoration was re-examined, this time using the criterion not sorted, for comparison. Both criteria were assessed for all restorations. The outcome variables were: indication of restorations replacement, indication of any type of treatment, and presence of caries.

### Setting

This study is nested in a clinical trial named Caries Cognition and Identification in Adults (CaCIA). The trial is registered on Clinicaltrials.gov under the number NCT03108586. Ethical approval was granted by the local Ethics Committee (protocol No. 1.625.236/2016).

The CaCIA trial is a randomized controlled clinical study that investigates the impact of the use of different visual criteria for the assessment of caries around restorations on the outcomes related to oral health in adults in the short and long term (1 to 5 years). The study was conducted at the School of Dentistry from the Federal University of Pelotas (Pelotas, Brazil).

The present study is part of a series of investigations conducted on the CaCIA clinical trial.

### Sample size

The sample used included all the patients from the randomized clinical trial CaCIA. Thus, 185 patients were included, and a total of 718 restorations were assessed. Details from the sample size calculation used on the clinical trial can be found on the protocol previously published [21].

### Participants—Eligibility criteria

Inclusion criteria:

1. patients seeking dental treatment at the School of Dentistry from the Federal University of Pelotas;
2. aged 18–60 years;
3. who presented at least one restoration (composite or amalgam) on a permanent posterior tooth.

Exclusion criteria:

1. patients who refused to participate in the research;
2. patients who presented systemic conditions or chronic diseases that require differentiated care and follow-up;
3. restorations on teeth with conditions such as fractures, fistula, abscess, pulp exposure, history of spontaneous dental pain, or mobility.

### Examiner training for restorations assessments

Previously to the study beginning, a training process was performed between one examiner (C.S) and one expert (M.S.C) in restorative dentistry and cariology, with extensive experience in restorations assessments and participation as a gold standard examiner in previous studies.

The training was divided in two phases. In the first phase, a series of photographs with restorations showing marginal defects was projected in a 50" HD television in a dark room for the examiner and the expert (gold standard). The examiner and expert discussed the evaluation and treatment of each restoration according to the FDI and CARS criteria.

The second phase was performed at the clinic. A total of 20 patients were assessed by the examiner and expert. They assigned the diagnosis and treatment according to FDI and CARS criteria for each case. The answers were compared in the end, and disagreements were discussed. A consensus was established.

### Interventions

The patients were examined in a dental chair under lighting. Before the assessment, they received a standard dental cleaning with low rotation micromotor, rubber cup, and brush with prophylactic paste. Initially, all patients' dental surfaces were examined according to the International Caries Detection and Assessment System (ICDAS) [13] for screening. The DMF-T (Decayed-Missing-Filled Teeth) index was also registered at this moment, as well as the patient caries activity.

The patients who met the inclusion criteria previously described were randomized by blocks considering subgroups based on the caries experience (DMF-T index less or equal to 4; or greater than 4) and caries activity (patient with or without active caries lesions). The participants were allocated into two groups according to the strategy to assess the restoratios:

- FDI criteria—based on 3 parameters from the FDI (International Dental Federation) system—caries presence, marginal adaptation and marginal staining (Table 1).
- CARS criteria—based on the "Caries Associated with Restorations or Sealants" (CARS) criteria described by the International Caries Classification and Management System (Table 2).

**Table 1:** Description of scores of FDI subcategories used to assess the restoration of this study

Subcategories	1 Clinically excellent	2 Clinically good	3 Clinically sufficient/satisfactory	4 Clinically unsatisfactory	5 Clinically poor
Marginal staining	No marginal staining	Minor staining, but easily removable by polishing	Moderate marginal staining, not esthetically unacceptable	Pronounced marginal staining; major intervention necessary for improvement	Deep marginal staining, not accessible for intervention
Marginal adaptation	Harmonious outline, no gaps, no white or discolored lines	Marginal gap (< 150 μm), white lines. Small marginal fracture removable by polishing. Slight ditching, slight step/flashes, minor irregularities. Gap < 250 μm not removable	Several small marginal fractures. Major irregularities ditching or flash, steps. Gap > 250 μm or dentine/base exposed	Severe ditching or marginal fractures. Larger irregularities or steps	Restoration (complete or partial) is loose but in situ. Generalized major gaps or irregularities
Recurrence of caries	No secondary or primary caries	Very small and localized demineralization	Larger areas of demineralization	Caries with cavitation	Deep secondary caries or exposed dentine that is not accessible for repair of restoration

The content of this table was based on the FDI criteria developed by International Dental Federation [22]

**Table 2:**
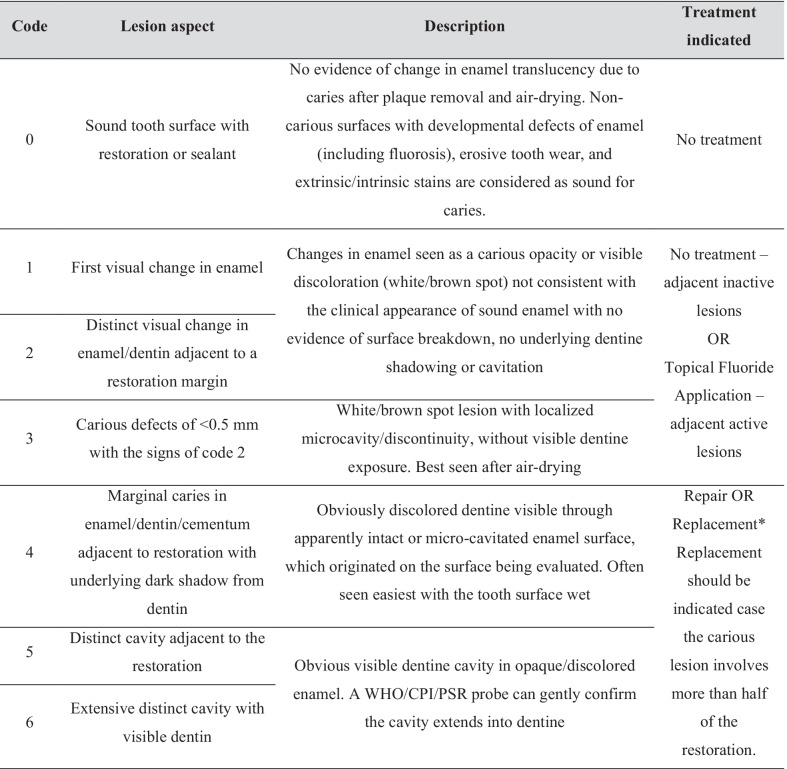
Lesion’s characteristics and treatment indication, respectively, of each CARS code based on CariesCare International 4D

The content of this table was based on the CARS criteria derived from ICDAS proposed by International Caries Classification and Management System (ICCMS) [23]

The restorations assessments was made using a dental mirror and a ball-point probe by the examiner. First, the examiner performed the assessment based on the randomized criteria and assigned the treatment according to the criteria recommendations. Following, the restorations were re-evaluated, but this time according to the other criteria. The same examiner assessed the restorations using both criteria to minimize other sources of variations.

- FDI criteria group

For the FDI assessment, all surfaces were dried before the evaluation [22]. Each one of the three aspects examined (caries presence, marginal adaptation and marginal staining) received a score from 1 to 5 for each restoration [1 = clinically excellent; 2 = clinically good; 3 = clinically sufficient/satisfactory, 4 = clinically unsatisfactory (but reparable), and, 5 = clinically poor (replacement necessary)] (Table 1). Restorations scored as 4 were indicated to be repaired, and those scores as 5 were indicated to be replaced. The highest score from the 3 aspects determined the final indication of treatment.

Concerning the assessment of amalgam restorations, taking into account the intrinsic pigmentation on tooth structure promoted by amalgam, only the aspects of marginal adaptation and caries recurrence were assessed. The assessment of marginal staining probably would end up in a score of number 5, which would lead to the replacement of, if not all the amalgam restorations, perhaps the majority of them.

The classification of the three aspects according to the FDI scores can be viewed in Table 1.

(b) CARS criteria group

CARS criteria are derived from ICDAS proposed by International Caries Classification and Management System (ICCMS) [23] and now updated to CariesCare International 4D [24]. The surfaces were evaluated wet and after being dried by 5 s with air [23]. Each restoration was assigned with a score ranging from 0 ('sound tooth surface with restoration') to 6 ('extensive distinct cavity with visible dentin'). The definition of each score and the treatment proposed for each one is presented in Table 2.

The treatment options for the included restorations were: (1) no treatment, (2) professional topical fluoride application, (3) refurbishment, (4) repair, and (5) replacement.

### Explanatory variables

The explanatory variables were divided into three different levels: the first level is related to the clinical evaluation considering the strategy used to assess the restorations (Criteria 1 and 2), and order of examination (the first criterion evaluated corresponds to the randomized one). The second level involves aspects related to the teeth, such as the type of teeth (molars and premolars), dental arch (upper or lower), number of restored surfaces (one surface, two surfaces, three or more surfaces), and restorative material (composite resin or amalgam). The third level is based on patients related variables. This level comprises sex, age (up to 30 years old or more than 30 years), DMF-T, and caries activity.

### Statistical analysis

Statistical analysis was conducted with statistical package Stata 13 (StataCorp LP, College Station, USA). Spearman's rank correlation analyses were performed between CARS and FDI scores. Marginal staining, marginal adaptation, and recurrence of caries were analyzed separately. For these, Spearman's correlation coefficient (Rho) and respective 95% CIs were calculated. 'Not evaluated' corresponding to marginal staining for amalgam restorations.

The treatment for restorations assessed by both criteria was classified into: (1) no treatment (restorations without treatment needs or those with an indication of topical fluoride application), (2) repair, or (3) replacement. Spearman's correlation analysis was conducted. Chi-square test was used adjusted by the cluster to compare the treatment decision between the FDI criteria and CARS.

Besides that, univariate and multiple Poisson multilevel regression analysis between primary outcome and explanatory variables were calculated, as also the PR (Prevalence Ratio) values and 95% CIs. First, univariate analyses was carried out. Then, a multiple regression analysis was conducted. For this analysis, the variables named diagnostic strategy, and dental material were inserted, regardless of the level of significance. Order of examinations was also included in all multiple models, to adjust the analysis considering a possible occurrence of incorporation bias, since the first method could exert an influence on the second method used by the examiner. Other variables with p-value < 0.05 were also maintained in the final model.

Similar Poisson multilevel regression analyses were also performed for the outcomes: any type of treatment and presence of caries lesions. The significance level was set at 5%.

## Results

A total of 185 patients were included in this study, from which 120 (65%) were female and 65 (35%) male. The patient's mean age was 41.8 years (SD = 15.8), with a range of 14 to 84 years old. And the DMF-T index mean was 11.4 (SD = 7.0), ranging from 1 to 29. Concerning the caries activity, 130 patients (70.2%) were assigned without caries activity, while 55 patients (29.8%) showed caries activity at the baseline assessment. According to the randomization, 90 (48.6%) patients were assessed by the CARS criteria, while 95 (51.4%) by the FDI criteria.

Teeth sample corresponded to 518 (72.1%) molars and 200 (27.9%) premolars. 345 (48%) teeth were located in the upper dental arch, while 373 (52%) teeth were at the lower dental arch. 55.8% had only one restored surface (n = 401), 29.8% 2 surfaces (n = 214) and 14.4% 3 or more surfaces (n = 103). The majority of the restorations (57.1%) were made of composite resin (n = 410), while 42.9% were made of amalgam (n = 308).

**Fig. 1:**
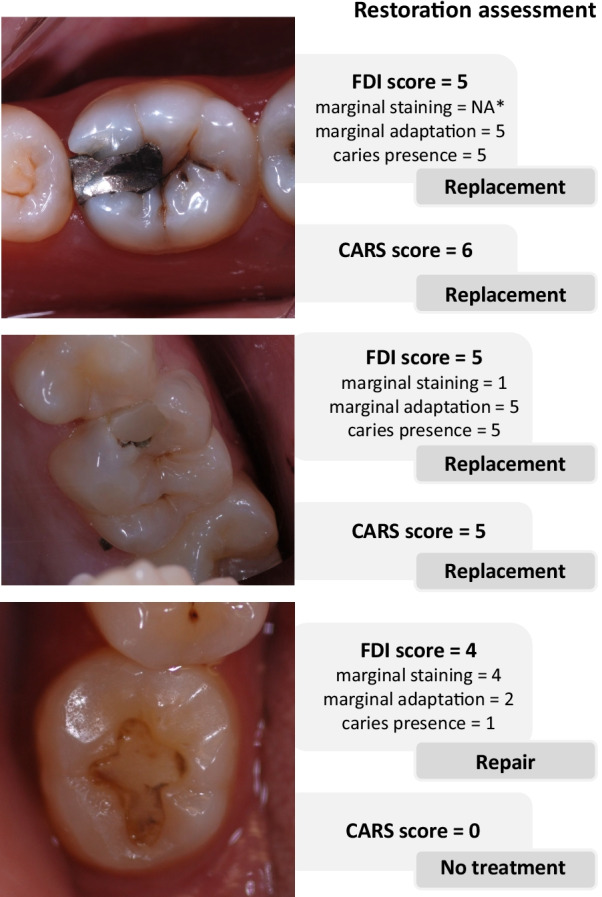
Illustrations of posterior restorations included in the study with the correspondent assessments (scores) and treatment decisions.*Note: NA = not applicable; marginal staining was not assessed on amalgam restorations

Figure 1 showns some of the posterior restorations included in the study with the correspondent assessments (scores) and treatment decisions for illustration.

Table 3 presents the correlation between the scores obtained using the FDI criteria and the CARS criteria. The more robust Spearman correlation coefficient (Rho) founded was related to the presence of caries lesions (Rho = 0.829). Marginal adaptation (Rho = 0.457) and marginal staining (Rho = 0.280) showed the lowest values.

**Table 3:** Spearman correlation coefficient (Rho) for CARS and FDI subcategories (marginal staining, marginal adaptation, and recurrence of caries) evaluated for included restorations

FDI criteria	CARS criteria							Total
	0	1	2	3	4	5	6	
*FDI marginal staining*								
1	57	1	12	1	6	7	0	84
2	52	2	22	11	6	8	3	104
3	33	0	43	26	8	15	0	125
4	20	2	29	7	10	5	0	73
5	6	0	6	6	8	2	1	29
Not evaluated	165	8	73	31	9	11	6	303

Rho = 0.280 (95% CI = 0.189–0.367)								
*FDI marginal adaptation*								
1	23	0	3	0	1	0	0	27
2	213	5	99	16	21	2	0	356
3	75	7	67	37	17	6	0	209
4	18	1	10	29	8	29	0	95
5	4	0	6	0	0	11	10	31

Rho = 0.457 (95% CI = 0.397–0.513)								
*FDI recurrence of caries*								
1	291	3	8	7	1	0	0	310
2	26	9	95	15	3	4	0	152
3	7	1	63	40	7	2	0	120
4	8	0	16	18	18	25	0	85
5	1	0	3	2	19	16	10	51

Rho = 0.829 (95% CI = 0.805–0.851)								
Total	333	13	185	82	47	48	10	718

Rho = Spearman correlation coefficient; 95% CI = 95% confidence interval

A moderate correlation (Rho = 0.420) was founded between the treatment decisions proposed by the CARS and by the FDI criteria (Table 4). Considering the 718 restorations evaluated, 16 restorations were suggested to be replaced (2.2%) when using the CARS criteria while the FDI criteria indicated the replacement of 83 restorations (11.6%), suggesting a more invasive approach. The CARS criterion led to 2 (0.28%) more invasive treatments compared to the FDI. More than 90% of the restorations assessed by the CARS criteria did not need operative treatment, while this number decrease to 66.4% for the restorations evaluated by the FDI.

**Table 4:** The relationship among treatment decisions indicated for assessment restorations comparing CARS and FDI criteria

FDI	CARS			Total
	No treatment	Repair	Replacement	
No treatment	476	0	1	477 (66.4%)
Repair	128	29	1	158 (22.0%)
Replacement	57	12	14	83 (11.6%)
Total	661 (92.1%)	41 (5.7%)	16 (2.2%)	718

Spearman correlation coefficient = 0.420 (95% Confidence interval = 0.358–0.478)

Chi-square adjusted by the cluster = 141.0; p < 0.001

The association among the explanatory variables and the restorations replacement is shown in Table 5. The multilevel regression analysis showed that the FDI criteria indicated five times more replacements when compared to CARS criteria (< 0.001). A positive association between indication for restorations replacement, with the DMF-T index (p = 0.031) and the number of restored surfaces (p < 0.010), was observed. No significant association between restorations replacements and the restorative material was found.

**Table 5:** Comparison between explanatory variables and the indications of restorations replacement (outcome) assessment by FDI and CARS criteria

Explanatory variables	Unadjusted PR (95%CI)	p	Adjusted PR (95%CI)	P
*Variables related to the patient (3rd level)*				
Sex (ref.: male)			*	
Female	1.41 (0.83–1.64)	0.206		
Age (ref.: up to 30 yrs-old)			*	
More than 30 yrs-old	1.01 (0.56–1.82)	0.978		
DMF-T (quant. variable)	1.04 (1.00–1.08)	0.034	1.04 (1.00–1.08)	0.031
Caries activity (ref.: no)				
Yes	1.35 (0.81–2.27)	0.251	1.62 (0.97–2.72)	0.067
*Variables related to the restored tooth (2nd level)*				
Type of teeth (ref.: Molars)			*	
Premolars	1.19 (0.75–1.88)	0.452		
Dental arch (ref.: upper)			*	
Lower	0.92 (0.61–1.41)	0.717		
Number of surfaces restored (ref.: 1 surface)				
2 surfaces	2.30 (1.42–3.72)	0.001	2.05 (1.25–3.37)	0.005
3 or more surfaces	2.60 (1.47–4.59)	0.001	2.19 (1.21–3.98)	0.010
Dental material (ref.: amalgam)				
Composite resin	1.66 (1.05–2.64)	0.031	1.42 (0.87–2.30)	0.157
*Variables related to the clinical evaluation (1st level)*				
Diagnostic method (ref.: CARS)				
FDI system	5.23 (3.07–8.93)	< 0.001	5.22 (3.05–8.91)	< 0.001
Order of examinations (ref.: 1st examination)				
2nd examination	1.02 (0.69–1.51)	0.904	0.95 (0.62–1.44)	0.809

PR = prevalence ratio; 95%CI = 95% confidence intervals; DMF-T = decayed, missed and filled permanent teeth

^*^Variables not included in the final model

When the outcome considered on the multilevel regression analysis was the indication of any type of treatment (Table 6), it was observed that active caries patients received 38% more indication for treatments (p = 0.012). The FDI criteria led to four times more interventions than the CARS criteria (p < 0.001). A higher number of interventions were recommended to the composite resin restorations compared to the amalgam restorations (p < 0.001). The restorations with two or more surfaces were also positively associated with a higher need of interventions recommended (p < 0.001).

**Table 6:** Comparison between explanatory variables and the indication of any type of treatment (outcome) assessment by FDI and CARS criteria

Explanatory variables	Unadjusted PR (95%CI)	p	Adjusted PR (95%CI)	P
*Variables related to the patient (3rd level)*				
Sex (ref.: male)			*	
Female	1.10 (0.82–1.48)	0.512		
Age (ref.: up to 30 yrs-old)			*	
More than 30 yrs-old	0.95 (0.68–1.32)	0.751		
DMF-T (quant. variable)	1.01 (0.99–1.03)	0.233	*	
Caries activity (ref.: no)				
Yes	1.39 (1.05–1.85)	0.023	1.38 (1.07–1.76)	0.012
*Variables related to the restored tooth (2nd level)*				
Type of teeth (ref.: Molars)			*	
Premolars	1.17 (0.91–1.51)	0.228		
Dental arch (ref.: upper)			*	
Lower	0.90 (0.71–1.14)	0.373		
Number of surfaces restored (ref.: 1 surface)				
2 surfaces	1.98 (1.51–2.60)	< 0.001	1.78 (1.35–2.33)	< 0.001
3 or more surfaces	3.14 (2.34–4.21)	< 0.001	2.58 (1.92–3.47)	< 0.001
Dental material (ref.: amalgam)				
Composite resin	2.52 (1.91–3.33)	< 0.001	1.96 (1.48–2.60)	< 0.001
*Variables related to the clinical evaluation (1st level)*				
Diagnostic method (ref.: CARS)				
FDI system	4.24 (3.18–5.66)	< 0.001	4.20 (3.15–5.61)	< 0.001
Order of examinations (ref.: 1st examination)				
2nd examination	1.13 (0.90–1.42)	0.280	1.12 (0.89–1.40)	0.331

PR = prevalence ratio; 95%CI = 95% confidence intervals; DMF-T = decayed, missed and filled permanent teeth

*Variables not included in the final model

Finally, Table 7 shows the comparison between the explanatory variables and the presence of caries assessed by the FDI and CARS criteria. The FDI criteria scored 2.7 times more frequently the restorations as having caries around when compared to the CARS criteria (p < 0.001). Besides, it was shown that restorations with three or more surfaces were more frequently scored as having a carious lesion when compared to a single surface restoration (p = 0.002). No statistically significant associations were identified between the restorative material and the presence of carious lesions, as well as the order of evaluation by the two criteria.

**Table 7:** Comparison between explanatory variables and the presence of caries (outcome) assessment by FDI and CARS criteria

Explanatory variables	Unadjusted PR (95%CI)	p	Adjusted PR (95%CI)	p
*Variables related to the patient (3rd level)*				
Sex (ref.: male)			*	
Female	0.98 (0.70–1.34)	0.843		
Age (ref.: up to 30 yrs-old)				
More than 30 yrs-old	0.74 (0.52–1.06)	0.098	0.83 (0.54–1.28)	0.403
DMF-T (quant. variable)	0.97 (0.95–1.00)	0.034	0.97 (0.95–1.00)	0.054
Caries activity (ref.: no)			*	
Yes	1.11 (0.79–1.57)	0.544		
*Variables related to the restored tooth (2nd level)*				
Type of teeth (ref.: Molars)			*	
Premolars	1.18 (0.85–1.63)	0.325		
Dental arch (ref.: upper)			*	
Lower	1.11 (0.82–1.51)	0.488		
Number of surfaces restored (ref.: 1 surface)				
2 surfaces	1.03 (0.72–1.47)	0.860	1.13 (0.78–1.62)	0.523
3 or more surfaces	1.65 (1.12–2.43)	0.011	1.89 (1.25–2.85)	0.002
Dental material (ref.: amalgam)				
Composite resin	1.02 (0.76–1.40)	0.857	0.81 (0.57–1.13)	0.215
*Variables related to the clinical evaluation (1st level)*				
Diagnostic method (ref.: CARS)				
FDI system	2.72 (1.93–3.83)	< 0.001	2.71 (1.93–3.81)	0.001
Order of examinations (ref.: 1st examination)				
2nd examination	1.14 (0.85–1.55)	0.383	1.12 (0.83–1.51)	0.474

PR = prevalence ratio; 95%CI = 95% confidence intervals; DMF-T = decayed, missed and filled permanent teeth

*Variables not included in the final model

## Discussion

This is the first clinical study to compare the use of two different clinical approaches for the detection of caries lesions around restorations in permanent teeth, and the effect on the treatment decision. The main findings of this study shows that the use of the FDI criteria (caries recurrence, marginal staining and adaptation) results in a higher number of interventions, mainly restorations, in comparison to the CARS criteria. Therefore, the strategy used to assess secondary caries may lead to different treatment decisions, more or less invasive. So, the study hypothesis was accepted.

The International Caries Detection and Assessment System (ICDAS) shows a list of well-described criteria for Caries Associated with Restorations and Sealants (CARS) [23]. Among the available criteria in the literature, the CARS criteria seem to be the most proper to be used nowadays. This criterion assesses the lesion severity and takes into account aspects not consistent with caries lesions, such as marginal staining and amalgam shadows. Also, the caries activity is also considered, with the evaluation of the presence of active enamel demineralization and cavities with soft tissues. The lesion activity influences the treatment decision for operative or non-operative treatment [25, 26]. Arrested lesions that allow hygiene, even if cavitated, will not necessarily require operative treatments. On the other hand, cavities in which it is not possible to access biofilm should be restored.

A strong positive correlation was observed between the criteria under investigation for the assessment of caries. This may be explained because the FDI criteria for caries recurrence relies on similar characteristics to those assessed by the International Caries Detection and Assessment System (ICDAS). It scales the lesion according to the lesion severity evaluating the presence of enamel opacities and dentine cavities, similar to the definitions adopted by CARS [22]. Nevertheless, the FDI criteria does not take into account the lesion activity. The addition of this aspect when using the ‘caries presence’ criterion from the FDI would probably improve this parameter on FDI system.

A moderate correlation was founded between CARS classification and the marginal adaptation from the restorations, assessed by the FDI criterion. This may be explained because although restorations with lack of adaptation, due to overhangs or gaps, are more prone to biofilm accumulation and caries lesions development, the absence of adaptation does not necessarily imply the development of caries lesions around restorations. The discrimination between the presence of gaps and caries lesions at the tooth-restoration interface is still a matter of divergences among clinicians and researchers [27, 28].

Also, a weak correlation was founded between the CARS criteria and the presence of marginal staining. The marginal staining is no longer understood as a factor related to the presence of secondary caries lesions [15, 29]. Even so, it is still considered one of the main aspects that lead to misinterpretations at the dental clinic [30], despite the evidence showing the staining as a poor predictor of caries [15, 29]. This is especially common in tooth-colored restorations in which brown and black marginal staining are misinterpreted as initial caries lesions. Besides, another factor that may have influenced the weak correlation was the absence of assessment of marginal staining in amalgam restorations. This is a limitation of this study. This decision was based on the fact that the majority of the amalgam restorations show an intrinsic pigmentation caused by the material on the dental structure [29]. So, we consider that probably an extremely high number of restorations would result in high scores for this aspect, indicating the replacement of the majority of the restorations. This possibly would lead to a significative overtreatment.

The multilevel regression analysis showed that the FDI criteria indicated five times more replacements when compared to the CARS criteria. Thus, the criterion used impacts directly on the decision to replace or not the restoration. A recent study from our group reported that on primary teeth, the decision to replace posterior restorations was influenced by the criteria used for the restorations assessment, as well by the children's caries experience and multisurface restorations [19]. The literature shows that more conservative approaches should be chosen considering the benefits for the patients, although this assertion is not based on a strong evidence [1, 5, 31].

It is important to note that a higher number of indications for intervention in the FDI group may be explained in part due to the inclusion of marginal staining and marginal adaptation as "caries related problems." In contrast, in the CARS criteria group, only the presence of caries lesion was considered. We adopted this approach because dental clinicians still use marginal defects as "markers" for caries around restorations and take treatment decisions based on these defects. Although evidence shows that marginal defects and pigmentations are not predictive factors for caries [17, 32, 33], they often are misinterpreted as secondary caries, leading to unnecessary clinical practice interventions. The secondary caries diagnosis process should rely only on the clinical (and in some cases radiographic) signs of the lesion, and not on the marginal characteristics of the restorations. Likewise, the secondary caries managing should be based on review/refurbishing/resealing/repairing instead of replacing partially defective restorations, as recently defined on an expert Delphi consensus statement [12].

In terms of clinical significance, the clinically relevant scores from FDI are scores 4 (repair) and 5 (replacement), when referring to the caries presence and marginal adaptation, because in these cases a restorative intervention is required most of the times. The scores 4 and 5 from FDI to marginal staining should be evaluated with attention as marginal staining alone in posterior teeth is not considered a clinically relevant problem. Regarding the CARS scores, scores 1, 2 and 3 will only be clinically relevant if the caries lesion around the restoration is in progress (active lesion). In theses cases topical fluoride application is indicated. And scores 4 up to 6 are also clinical significant because they are related to the need of repair or replacement of restorations.

The restoration size (3 or more surfaces) proved to be a significant factor in the three outcomes evaluated (the indication of replacement, any treatment, and the presence of caries). Other studies [34, 35] already showed major failures in extensive restorations, in agreement with the higher indication of treatment for this type of restoration in this study. Other variables such as caries activity, DMF-T index, and restoration material also showed to influence the treatment indication, in agreement with published studies [6, 7, 30, 36–38].

Regarding the restorative material, when the outcome 'any type of treatment' was analyzed, more interventions were recommended to the composite resin restorations compared to the amalgam restorations. One hypothesis to explain this finding is probably the higher indication for the repair of resin restorations compared to the amalgam ones. Some studies show the higher development for caries lesions around restorations on composite resin restorations compared to the amalgam ones [8, 39–41]. On the other hand, there is also evidence that the material used does not influence caries lesion development [42]. It should be noted that, the material used on the restoration has a small role in the longevity of the restoration [38]. The factors related to the patient, such as the caries risk, have a major role. The caries prevention depends basically on oral hygiene and dietary habits, which is essential to decrease the incidence of caries around restorations and prolong the longevity of the restorations [12].

A limitation of the study was the use of only one examiner to assess both criteria on the restorations. However, to minimize this limitation the order of the criteria examination was included as a variable related to the clinical evaluation on the multilevel regression analysis. This inclusion aimed to build a reliable model. The examination order showed no statistically significant difference among the three outcomes evaluated, which may indicate that there was no bias in the restoration evaluation by the examiner according to the randomized criteria. Besides, this ratifies the study calibration process. We also belief that the inclusion of more examiners would result on other possible fonts of variations. So, just one examiner executed the assessments, following exactly the objective description of the criteria. Other study limitation is the lack of a method to measure the ability of the examiner to record the same conditions the same way over time. However, the use of a well described criteria based on a scoring system with a detailed description it seems capable to corroborate to the intraexaminer reproducibility. Standardized measures are used to minimize measurements variations. Also, it was already shown that the intraexaminer reliability to caries detection is high and remains high over the time [43].

Nevertheless, although the FDI criteria appears to be less conservative, indicating a higher level of restorations replacement than CARS, and probably ending up in overtreatment, it is still not possible to state through a cross-sectional study which is the best criterion for the evaluation of restorations. This question will only be answered by the ongoing clinical trial mentioned in this study. Further studies evaluating the influence of the diagnostic methods on dental treatment decisions should be performed to implement an evidence-based dentistry. Moreover, this study was not designed to compare the FDI criteria entirely, developed to assess dental restorations, with the CARS criteria, which was produced to assess secondary caries lesions only. The study was assembled to examine different possible clinical approaches that can be adopted for secondary caries diagnosis and management, and the interpretation of the results should be limited to this scope.

## Conclusions

In conclusion, the visual criteria used on the restoration's assessment directly influences the treatment decision to intervene or not on the restoration. The use of a minimally invasive based approach for assessing secondary caries may prevent overtreatment.

## Supplementary Information

**Additional file 1.** STROBE checklist.

## Data Availability

The datasets generated and/or analysed during the current study are not publicly available yet. The datasets will be available in a public repository after the acceptance of the manuscripts using the data derived from the clinical trial previously mentioned. However, data are available from the corresponding author on reasonable request.

## Abbreviations

CaCIA: Caries Cognition and Identification in Adults
CARDEC: CARies DEection in Children
CARS: Caries Associated with Restorations or Sealants
CI: Confidence Interval
DMF-T: Decayed, Missing, Filled permanent teeth
FDI: World Dental federation
ICCMS: International Caries Classification and Management System
UFPEL: Federal University of Pelotas

## References

[1] Sheiham A (2002). Minimal intervention in dental care. Med Princ Pract.

[2] Henry DB (2009). The consequences of restorative cycles. Oper Dent.

[3] Henry DB (2014). The restorative cycle in dentistry. Todays FDA.

[4] Wilson N, Lynch C, Brunton P, Hickel R, Meyer-Lueckel H, Gurgan S (2016). Criteria for the replacement of restorations: academy of operative dentistry European section. Oper Dent.

[5] Deligeorgi V, Mjör IA, Wilson NH (2001). An overview of reasons for the placement and replacement of restorations. Prim Dent Care.

[6] Pallesen U, Van DJWV, Halken J, Hallonsten A-L, Höigaard R (2014). A prospective 8-year follow-up of posterior resin composite restorations in permanent teeth of children and adolescents in Public Dental Health Service: reasons for replacement. Clin Oral Investig.

[7] Van de Sande FH, Opdam NJ, Rodolpho PADR, Correa MB, Demarco FF, Cenci MS (2013). Patient risk factors ’ influence on survival of posterior composites. J Dent Res.

[8] Nedeljkovic I, De Munck J, Vanloy A, Declerck D, Lambrechts P, Peumans M (2019). Secondary caries: prevalence, characteristics, and approach. Clin Oral Investig.

[9] Mjör IA, Toffenetti F (2000). Secondary caries: a literature review with case reports. Quintessence Int.

[10] Askar H, Krois J, Göstemeyer G, Bottenberg P, Zero D, Banerjee A (2020). Secondary caries: what is it, and how it can be controlled, detected, and managed?. Clin Oral Investig.

[11] Seemann R, Flury S, Pfefferkorn F, Lussi A, Noack MJ (2014). Restorative dentistry and restorative materials over the next 20 years: a Delphi survey. Dent Mater.

[12] Schwendicke F, Splieth CH, Bottenberg P, Breschi L, Campus G (2020). How to intervene in the caries process in adults : proximal and secondary caries? An EFCD-ORCA-DGZ expert Delphi consensus statement. Clin Oral Investig.

[13] Signori C, Gimenez T, Mendes FM, Huysmans MCDNJM, Opdam NJM, Cenci MS (2018). Clinical relevance of studies on the visual and radiographic methods for detecting secondary caries lesions—a systematic review. J Dent.

[14] Mjor IA (2005). Clinical diagnosis of recurrent caries. J Am Dent Assoc.

[15] Kidd EA (2001). Diagnosis of secondary caries. J Dent Educ.

[16] Kidd EAM, Beighton D (1995). Marginal ditching and staining as a predictor of secondary caries around amalgam restorations: a clinical and microbiological study. J Dent Res.

[17] Kidd EAM, Beighton D (1996). Prediction of secondary caries around tooth-colored restorations: a clinical and microbiological study. J Dent Res.

[18] Schwendicke F, Splieth CH, Bottenberg P, Breschi L, Campus G, Doméjean S (2020). How to intervene in the caries process in adults: proximal and secondary caries? An EFCD-ORCA-DGZ expert Delphi consensus statement. Clin Oral Investig.

[19] Moro BLP, Freitas RD, Pontes LRA, Pássaro AL, Lenzi TL, Tedesco TK (2020). Influence of different clinical criteria on the decision to replace restorations in primary teeth. J Dent.

[20] Von Elm E, Altman DG, Egger M, Pocock SJ, Gøtzsche PC, Vandenbrouckef JP (2007). The Strengthening the Reporting of Observational Studies in Epidemiology (STROBE) Statement: guidelines for reporting observational studies. Bull World Health Organ.

[21] Signori C, Moro BLP, Uehara JLS, Romero VHD, de Oliveira EF, Braga MM (2020). Study protocol for a diagnostic randomized clinical trial to evaluate the effect of the use of two clinical criteria in the assessment of caries lesions around restorations in adults: the Caries Cognition and Identification in Adults (CaCIA) trial. BMC Oral Health.

[22] Hickel R, Peschke A, Tyas M, Mjör I, Bayne S, Peters M (2010). FDI World Dental Federation—clinical criteria for the evaluation of direct and indirect restorations. Update and clinical examples. J Adhes Dent.

[23] Pitts NB, Ekstrand K (2013). International caries detection and assessment system (ICDAS) and its international caries classification and management system (ICCMS) - Methods for staging of the caries process and enabling dentists to manage caries. Community Dent Oral Epidemiol.

[24] Martignon S, Pitts NB, Goffin G, Mazevet M, Douglas GVA, Newton JT (2019). CariesCare practice guide: consensus on evidence into practice. Br Dent J.

[25] Kidd E (2011). The implications of the new paradigm of dental caries. J Dent.

[26] Dennison JB, Sarrett DC (2012). Prediction and diagnosis of clinical outcomes affecting restoration margins. J Oral Rehabil.

[27] Maske TT, Kuper NK, Cenci MS, Huysmans MCDNJM (2017). Minimal gap size and dentin wall lesion development next to resin composite in a microcosm biofilm model. Caries Res.

[28] Kuper NK, Opdam NJM, Ruben JL, de Soet JJ, Cenci MS, Bronkhorst EM (2014). Gap size and wall lesion development next to composite. J Dent Res.

[29] Kidd EAM, Joyston-Bechal S, Beighton D (1995). Marginal ditching and staining as a predictor of secondary caries around amalgam restorations: a clinical and microbiological study. J Dent Res.

[30] Demarco FF, Collares K, Correa MB, Cenci MS, de Moraes RR, Johannes ON (2017). Should my composite restorations last forever? Why are they failing?. Braz Oral Res.

[31] Moncada G, Vildósola P, Fernández E, Estay J, De Oliveira Júnior OB, De Andrade MF (2015). Longitudinal results of a 10-year clinical trial of repair of amalgam restorations. Oper Dent.

[32] Kidd EA, Joyston-Bechal S, Beighton D (1994). Diagnosis of secondary caries: a laboratory study. Br Dent J.

[33] Foster LV (1994). Validity of clinical judgements for the presence of secondary caries associated with defective amalgam restorations. Br Dent J.

[34] Opdam NJM, Van De Sande FH, Bronkhorst E, Cenci MS, Bottenberg P, Pallesen U (2014). Longevity of posterior composite restorations: a systematic review and meta-analysis. J Dent Res.

[35] da Rosa Rodolpho PA, Cenci MS, Donassollo TA, Loguércio AD, Demarco FF (2006). A clinical evaluation of posterior composite restorations: 17-year findings. J Dent.

[36] Bucher K, Metz I, Pitchika V, Hickel R, Kuhnisch J (2015). Survival characteristics of composite restorations in primary teeth. Clin Oral Investig.

[37] van de Sande F, Collares K, Correa M, Cenci M, Demarco F, Opdam N (2016). Restoration survival: revisiting patients’ risk factors through a systematic literature review. Oper Dent.

[38] Demarco FF, Corrêa MB, Cenci MS, Moraes RR, Opdam NJM (2012). Longevity of posterior composite restorations: not only a matter of materials. Dent Mater.

[39] Soncini JA, Maserejian NN, Trachtenberg F, Tavares M, Hayes C (2007). The longevity of amalgam versus compomer/composite restorations in posterior primary and permanent teeth findings from the New England children’s Amalgam trial. J Am Dent Assoc.

[40] Bernardo M, Luis H, Martin MD, Leroux BG, Rue T, Leitão J (2007). Survival and reasons for failure of amalgam versus composite posterior restorations placed in a randomized clinical trial. J Am Dent Assoc.

[41] Nedeljkovic I, Teughels W, De Munck J, Van Meerbeek B, Van Landuyt KL (2015). Is secondary caries with composites a material-based problem?. Dent Mater.

[42] Opdam NJM, Bronkhorst EM, Loomans BAC, Huysmans MCDNJM (2010). 12-Year survival of composite vs. amalgam restorations. J Dent Res.

[43] Qudeimat MA, Alomari QD, Altarakemah Y, Alshawaf N, Honkala EJ (2016). Variables affecting the inter- and intra-examiner reliability of ICDAS for occlusal caries diagnosis in permanent molars. J Public Health Dent.

